# Characterization of cellular senescence mechanisms in human corneal endothelial cells

**DOI:** 10.1111/j.1474-9726.2011.00776.x

**Published:** 2012-04

**Authors:** Angela N Sheerin, S Kaye Smith, Katrin Jennert-Burston, Amy J Brook, Marcus C Allen, Badr Ibrahim, Dawn Jones, Corrin Wallis, Katrin Engelmann, William Rhys-Williams, Richard G A Faragher, David Kipling

**Affiliations:** 1 School of Pharmacy and Biomolecular Sciences, University of BrightonHuxley Building, Lewes Road, Brighton BN2 4GJ, UK; 2 School of Medicine, Cardiff UniversityHeath Park, Cardiff CF14 4XN, UK; 3 Department of Ophthalmology, Klinikum Chemnitz GmbH, Klinik für Augenheilkunde, Flemmingstraße 209116 Chemnitz, Dresden, Germany; 4 DFG-Center for Regenerative Therapies DresdenTatzberg 47/49, D-01307 Dresden, Germany; 5 Destiny Pharma Ltd., Sussex Innovation Centre, Science Park Square, FalmerBrighton BN1 9SB, UK

**Keywords:** senescence, telomeres, telomerase, p53, oxidative stress, CDK4, replicative senescence

## Abstract

The human cornea is a tri-laminar structure composed of several cell types with substantial mitotic potential. Age-related changes in the cornea are associated with declining visual acuity and the onset of overt age-related corneal diseases. Corneal transplantation is commonly used to restore vision in patients with damaged or diseased corneas. However, the supply of donor tissue is limited, and thus there is considerable interest in the development of tissue-engineered alternatives. A major obstacle to these approaches is the short replicative lifespan of primary human corneal endothelial cells (HCEC). Accordingly, a comprehensive investigation of the signalling pathways and mechanisms underpinning proliferative lifespan and senescence in HCEC was undertaken. The effects of exogenous human telomerase reverse transcriptase expression, p53 knockdown, disruption of the pRb pathway by over-expression of CDK4 and reduced oxygen concentration on the lifespan of primary HCEC were evaluated. We provide proof-of-principle that forced expression of telomerase, when combined with either p53 knockdown or CDK4 over-expression, is sufficient to produce immortalized HCEC lines. The resultant cell lines express an HCEC-specific transcriptional fingerprint, and retain expression of the corneal endothelial temperature-sensitive potassium channel, suggesting that significant dedifferentiation does not occur as a result of these modes of immortalization. Exploiting these insights into proliferative lifespan barriers in HCEC will underpin the development of novel strategies for cell-based therapies in the human cornea.

## Introduction

Structurally, the cornea has three layers, each populated by a different cell type with highly distinctive functions and patterns of gene expression (Joyce, 2003). The outermost epithelium is a self-renewing multilayered epithelial sheet. Located beneath the epithelium, the stroma is a structured lattice of collagens and proteoglycans deposited and maintained by specialized keratocytes. Below the stroma, the endothelium is a single layer of cells on the innermost surface that forms the boundary between the cornea and the aqueous humour. Corneal endothelial cells have a number of essential functions including active transport of nutrients from the aqueous humour into the stroma and regulation of stromal hydration via Na^+^/K^+^ ATPase pumps to maintain corneal transparency (Fischbarg & Maurice, 2004). *In vivo* human corneal endothelial cells (HCEC) reside within the highly growth-factor-depleted environment of the aqueous humour, and rarely enter mitosis under normal conditions (Klenkler & Sheardown, 2004). Thus, in many species, the repair of wounds to the corneal endothelium is principally achieved by cell enlargement and migration rather than cell division (Joyce, 2003). This quiescent state is thought to be maintained *in vivo* by a combination of contact inhibition and antiproliferative autocrine and paracrine TGFβ signalling (Joyce, 2003).

**Fig. 1:**
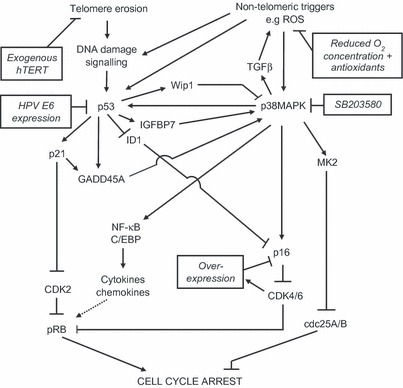
Pathways involved in establishment of senescence cell cycle arrest. Telomeric and environmental cues trigger senescence-associated cell cycle arrest via pathways regulated by the tumour suppressors p53 and pRb. Growth arrest can be reinforced by autocrine signalling loops modulated by NF-κB and C/EPB (Adams, 2009), and feedback via p21-p38MAPK-TGFβ and ROS (Passos *et al.*, 2010). These pathways can be manipulated to delay or prevent onset of senescence by the experimental interventions in the boxes. The figure shows selected pathways and is simplified for clarity. ROS, reactive oxygen species.

Despite their proliferation being severely restricted *in vivo*, HCEC retain the ability to divide when removed from this environment, and primary cultures can be established from explants of donor corneal tissue (Engelmann *et al.*, 1988; Kipling *et al.*, 2009). However, after a relatively short proliferative lifespan [typically 20–30 population doublings (PD)], HCEC cultures enter senescence. Senescence is a process at the single cell level that acts to limit excessive cell division and halt early neoplastic progression (Adams, 2009). It is characterized by long-term cell cycle arrest accompanied by altered cellular morphology and physiology (Campisi & d’Adda di Fagagna, 2007; Adams, 2009). Senescence is regulated in a cell type- and stimulus-dependent manner by a complex signalling network involving the tumour suppressors, p53 and pRb (Fig. 1). Expression of exogenous human telomerase reverse transcriptase (hTERT) in several human cell types restores telomerase activity and prevents replication-dependent telomere erosion (Bodnar *et al.*, 1998; Vaziri & Benchimol, 1998), thus preventing the telomere-dependent DNA damage signal that induces senescence in certain cell types (d’Adda di Fagagna *et al.*, 2003). As a result, expression of hTERT leads to immortalization with retention of normal physiological function in many human cell types including fibroblasts, bronchial epithelial cells and corneal epithelial cells, and is therefore considered to be a promising tool for boosting cell numbers in bioengineering applications (Robertson *et al.*, 2005; Shay & Wright, 2005).

However, several human cell types including myoblasts (Zhu *et al.*, 2007), skin keratinocytes (Kiyono *et al.*, 1998) and astrocytes (Evans *et al.*, 2003) do not show extension of cellular lifespan following forced expression of hTERT. These data show that substantial cell type specificity exists in the senescence mechanisms and regulators used, and that the mechanisms underlying replicative senescence in any given cell type cannot be assumed but must be characterized on a cell type by cell-type basis (Shay & Wright, 2005).

*In vivo* studies provide some clues to the potential senescence mechanisms operating within HCEC. Donor age and anatomical location within the corneal endothelium influence the capacity of explanted HCEC to divide *ex vivo* (Zhu & Joyce, 2004; Enomoto *et al.*, 2006; Mimura & Joyce, 2006), suggesting the presence of senescent cells in human corneal endothelium *in vivo*. The expression of genes associated with senescence, such as the cyclin-dependent kinase inhibitors (CDKIs) p16^INK4a^ and p21^CIP1^, has been reported to be increased in the corneal endothelium, and primary HCEC cultures derived from them, of donors over the age of fifty years (Enomoto *et al.*, 2006; Song *et al.*, 2008). These increases in CDKIs are reflected in slower kinetics of pRb hyperphosphorylation in primary HCEC from older donors (Enomoto *et al.*, 2006). In addition, SA-βgal staining, a marker of senescence, was absent in corneal endothelium of young donors but present in approximately 50% of peripheral cells and 80% of central cells in corneal endothelium of donors over fifty (Mimura & Joyce, 2006; Song *et al.*, 2008). However, these differences in replicative capacity and expression of senescence markers between younger and older donors, and peripheral vs. central HCEC, were not associated with differences in telomere length. This observation was interpreted as indicating that senescence may be induced in corneal endothelium *in vivo* by telomere-independent mechanisms (Konomi & Joyce, 2007).

Whilst it is possible that senescent cells may contribute to age-related ocular pathologies (Faragher *et al.*, 1997b), it is clear that there is a practical need to understand the proliferative lifespan barriers operating within HCEC *in vitro* to manufacture utile cell lines for both fundamental study and translational use. Accordingly, we have carried out a detailed dissection of the molecular mechanisms that control replicative senescence (Fig. 1) in cultured HCEC, and have established the routes by which these may most efficiently be bypassed so as to support the future development of functional, differentiated endothelial cell lines.

## Results

### Late passage HCEC show morphological features of senescence and a transcriptome enriched with p53 targets, but lack elevated CDKIs

**Fig. 2:**
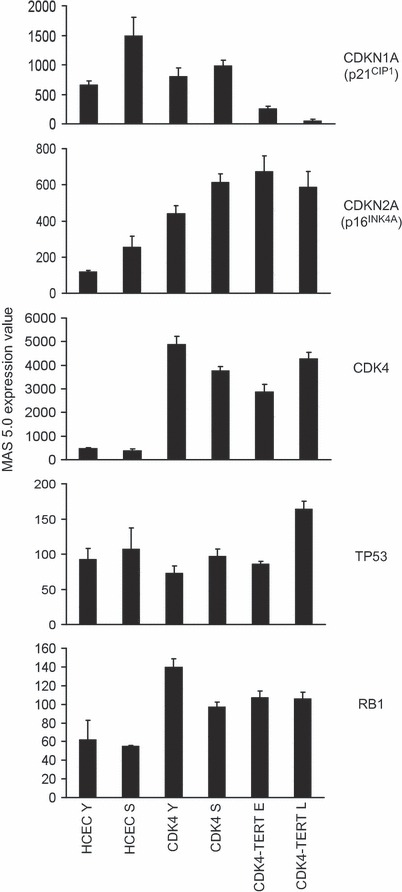
Expression of cell cycle and senescence-associated gene transcripts. Transcript abundance was measured on Affymetrix HG-U133A arrays and analysed using Microarray Suite 5.0 (MAS 5; Affymetrix, Santa Clara, CA, USA). Cell samples are annotated as follows: PD, population doublings; human corneal endothelial cells (HCEC) Y, proliferating young HCEC (10 PD); HCEC S, senescent HCEC (29 PD); CDK4 Y, proliferating HCEC-CDK4 (38 PD); CDK4 S, senescent HCEC-CDK4 (56 PD); CDK4-TERT, immortal ‘Zante’ HCEC-CDK4-hTERT line, E, early passage (53 PD), L, late passage (193 PD). Values are average of three experiments. Error bars represent standard deviation. hTERT, human telomerase reverse transcriptase.

**Fig. 3:**
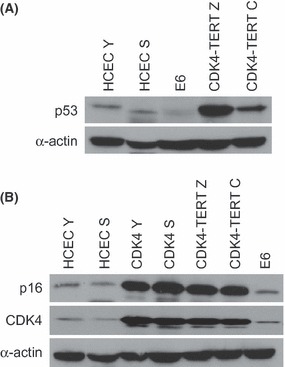
Western blot analysis of cell cycle and senescence-associated proteins. Effects of senescence, E6, or CDK4 plus human telomerase reverse transcriptase (hTERT) expression on (a) p53 levels and (b) CDK4 and p16 expression. Cell samples are annotated as follows: human corneal endothelial cells (HCEC) Y: proliferating young HCEC (10 PD); HCEC S, senescent HCEC (22 PD); E6, HCEC expressing HPV16 E6; CDK4-TERT Z, HCEC-CDK4-hTERT (Zante line); CDK4-TERT C, HCEC-CDK4-hTERT (Cardiff line); CDK4 Y, proliferating HCEC-CDK4 (38 PD); CDK4 S, senescent HCEC-CDK4 (56 PD).

Strains of HCEC will proliferate for approximately 20 PD in culture (Fig. S1). At the end of this proliferative lifespan, the cultures are overwhelmingly composed of cells that show characteristics typical of replicative senescence. These include an enlarged, flattened morphology and lack of proliferation (as measured by Ki67 staining) when compared with early passage HCEC (data not shown). A comparison of the transcriptomes of these ‘senescent’ HCEC with their proliferating and quiescent isogenic counterparts showed that a cluster of genes differentially up-regulated in senescent HCEC is significantly enriched with p53 transcriptional targets (Table S1, Fig. S2), although p53 itself is not up-regulated at the protein or transcript level (Figs 2 and 3), consistent with previous studies of fibroblast senescence (Webley *et al.*, 2000). An up-regulation of p21^CIP1^ (CDKN1A) and p16^INK4A^ (CDKN2A) at the transcript level is detectable in senescent HCEC (Fig. 2), although marked changes at the protein level at senescence are absent (Fig. 3b, lanes 1 and 2 and data not shown).

### Senescence in HCEC is not driven by telomere length or stress signalling but is modulated by extrinsic factors that impact upon oxidative stress

**Fig. 4:**
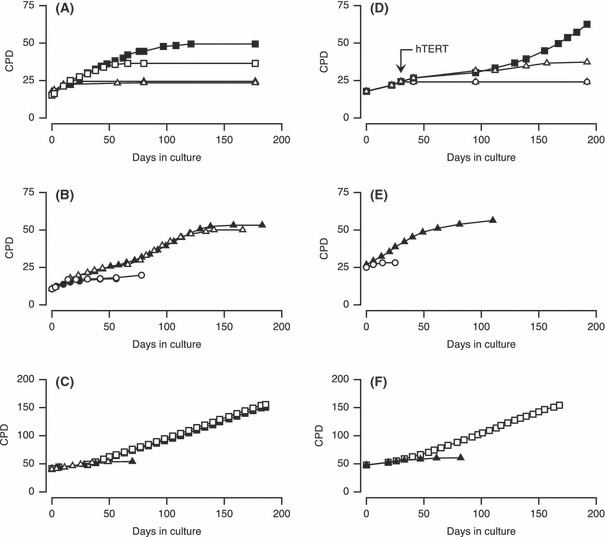
Experimental intervention and human corneal endothelial cells (HCEC) lifespan. (a) Expression of exogenous human telomerase reverse transcriptase (hTERT) extends proliferative lifespan of HCEC cultured in 3% O_2_ with ascorbic acid 2-phophate. (Δ) vector control 20% O_2_; (▴) HCEC-hTERT 20% O_2_; (□) vector control 3% O_2_ plus ascorbic acid 2-phosphate; (▪) HCEC-hTERT 3% O_2_ plus ascorbic acid 2-phosphate. (b) Extension of HCEC lifespan by expression of HPV16 E6. (•) normal HCEC; (○) HCEC-vector control; (Δ) HCEC-E6 culture 1; (▴) HCEC-E6 culture 2. (c) Combined expression of HPV16 E6 and hTERT immortalizes HCEC. (Δ) HCEC-E6; (▴) HCEC-E6-pBABE; (□) HCEC-E6-hTERT culture 1; (▪) HCEC-E6-hTERT culture 2. (d) Effects of p53 knockdown by shRNA and expression of hTERT. (○) vector control HCEC-pMKO.1PS; (Δ) HCEC-p53sh-pBABEneo; (▪) HCEC-p53sh-hTERT. (e) Bypass of senescence by forced expression of CDK4. (○) vector control; (▴) HCEC-CDK4. (f) Immortalization of HCEC by forced expression of CDK4 in combination with hTERT. (▴) HCEC-CDK4-pBABEpuro; (□) HCEC-CDK4-hTERT. CPD, cumulative population doublings.

Figure 4 shows the effects of stabilization of telomere length in primary HCEC by ectopic expression of hTERT using a pBABE series retroviral vector under standard culture conditions. No extension of replicative lifespan was observed either by mass culture or in individual clones. However, reducing the partial pressure of oxygen in the culture incubator to 3% and adding ascorbic acid 2-phosphate to the growth medium produced a significant increase (12 PD) in the capacity of HCEC to proliferate *in vitro*. Expression of hTERT under these conditions extends replicative lifespan to 50 PD but does not produce immortalization. Blockade of p38MAP kinase signalling in primary HCEC by treatment with the inhibitor SB203580 did not alter their lifespan, despite control experiments with anisomycin-stimulated HCEC that confirmed that this compound was able to block stress signalling (Fig. S3).

### Telomerase expression in combination with the ablation of p53 function will immortalize HCEC

Ectopic expression of the E6 oncoprotein of human papilloma virus 16 (HPV16) in HCEC using a pBABE series retroviral vector (Bond *et al.*, 1999; Davis *et al.*, 2003; Evans *et al.*, 2003) reduces p53 protein expression as measured by western blotting (Fig. 3a, Fig. S4) and extends the replicative lifespan of primary HCEC by approximately 30 PD (Fig. 4b). Similarly, infection of HCEC with a retroviral vector carrying a small hairpin RNA directed against p53 produced a significant extension of lifespan to 37 PD, at which point the culture ceased to expand (Fig. S4, Fig. 4d). Reinfection of E6- or p53 shRNA-expressing cells with a second retroviral vector expressing hTERT prevented this growth arrest and resulted in extensive proliferation (Fig. 4c,d). HCEC cell lines coexpressing E6 and hTERT have now proliferated at least 100 PD beyond the replicative limit of the progenitor primary HCEC and may be considered immortal.

### Ectopic expression of CDK4 can also immortalize HCEC in the presence of telomerase

Introduction of a wild-type CDK4 transgene into HCEC produced elevated levels of CDK4 protein (Fig. 3b, lanes 3 and 4) and in turn elevated p16^INK4a^ at both the protein (Fig. 3b) and transcript (Fig. 2) levels, consistent with a known feedback circuit (Ruas *et al.*, 2007), and elevated transcript abundance (Fig. S5a,b) of E2F targets (Blais & Dynlacht, 2004). Over-expression of CDK4 extended HCEC proliferative lifespan by 28 PD (Fig. 4e) and introduction of hTERT either at the same time as CDK4 (denoted ‘Zante’; Fig. S5c) or else subsequent to CDK4 (denoted ‘Cardiff’; Fig. 4f) gave further extension of lifespan to produce cell lines that have undergone at least 100 PD more than primary HCEC, and are thus judged to be immortal. pRb, p53 and p16^INK4A^ remained detectable at both transcript and protein level (Figs 2 and 3 and data not shown) in both cell lines, as was elevated CDK4 expression (Fig. 3b).

### Immortalized HCEC retain the transcriptional signatures and characteristics typical of primary cells

**Fig. 5:**
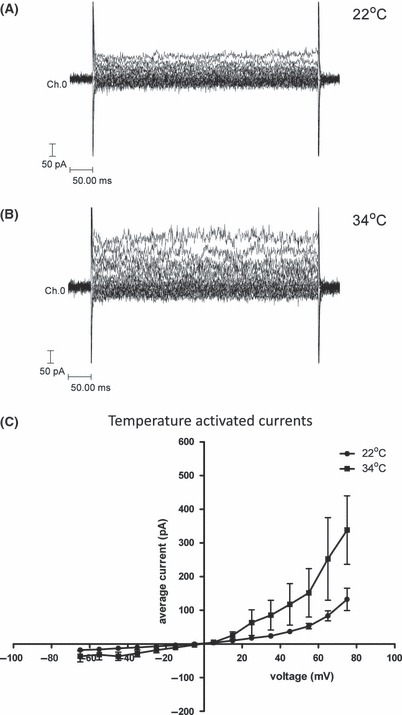
Expression of a temperature-sensitive potassium channel in human corneal endothelial cells (HCEC) immortalized with human telomerase reverse transcriptase (hTERT) and CDK4. HCEC-CDK4/TERT cells (Zante at beyond PD 100) were used in these experiments. A single cell was voltage clamped at −5 mV and voltage gated macroscopic currents recorded at (a) 22 °C and (b) 34 °C by stepping the potential between −65 and 75 mV. An outward rectifying potassium current is activated in these cells by raising the temperature as illustrated by the current voltage plot in (c) (*n* = 4).

Both the Zante and Cardiff cell lines have retained normal growth controls, as judged by retaining their ability to exit from the cell cycle in response to contact inhibition (data not shown). To determine whether any substantial alteration in corneal endothelial phenotype had occurred in the cell lines, the expression profiles of primary HCEC, the CDK4-hTERT Zante cell line and three other primary cell strains (Ek1.Br corneal keratocytes, together with HCA2 and WI38 primary human fibroblasts) were obtained using Affymetrix GeneChip microarrays. Clustering analysis of differentially expressed genes, defined using anova, showed that the Zante cell line data clustered closely with primary HCEC and separately from the other three cell strains (Fig. S6a). These data were supplemented using a set of thirty-three genes that exhibit high expression levels in HCEC relative to each of the other cell types (Kipling *et al.*, 2009), and thus constitute an HCEC-specific transcriptional fingerprint. Expression levels of these genes by two cultures of the Zante cell line at different cumulative PD levels was examined and compared with expression of the same transcripts in HCEC, EK1.Br, HCA2 and WI38 (Fig. S6b and Table S2). HCEC-CDK4-TERT Zante cells at the earlier PD level of 52 retained elevated expression of at least 25 of the 33 genes differentially up-regulated in primary HCEC, thus maintaining a very similar profile to the parent primary HCEC strain. The transcriptome data also revealed that the cell lines retain mRNA-level expression of COL8A2, a major component of Descemet‘s membrane underlying the corneal endothelium and a marker for HCEC (Engler *et al.*, 2009), and the corneal endothelial cotransporters SLC4A4, SLC4A2, SLC9A6 and NKCC1 (Bonanno, 2011). In addition, whole-cell patch-clamp measurements on the Zante cell line demonstrated the presence of a temperature-sensitive potassium channel (Fig. 5), together with dual expression of the intermediate filament proteins cytokeratin and vimentin (data not shown).

## Discussion

Although cellular senescence has been widely studied in numerous cell types, before the current study little was known about pathways to senescence in HCEC. The data we have presented provide the most comprehensive description to date of the regulation of cellular lifespan in this human cell type.

It is clear from our study that HCEC exit from the cell cycle and enter a state of viable proliferative arrest consistent with replicative senescence. Senescence in this cell type is associated with up-regulation of a cluster of genes that is significantly enriched with p53 transcriptional targets. p53 message and protein levels themselves do not change, consistent with the previous studies in other cell types demonstrating that the activation of p53 in senescence occurs primarily via post-translational modifications (Webley *et al.*, 2000). Similarly, although up-regulation of p21^CIP1^ and p16^INK4A^ at the transcript level is detectable in these senescent HCEC, this is not reflected to the same extent at the protein level. This is consistent with a recent report that p21^CIP1^ expression is controlled separately at the transcriptional and translational levels in human fibroblast senescence and that elevated p21^CIP1^ transcription does not simply translate into enhanced p21^CIP1^ protein (Lian & Gallouzi, 2009).

The available *in vivo* data on CDKI expression in HCEC, although limited by small sample numbers, is consistent with an elevation of p16^INK4a^ at both protein and transcript levels in HCEC from older donors (Enomoto *et al.*, 2006; Song *et al.*, 2008). The situation with p21^CIP1^ is less clear, with apparent elevation at the protein (Enomoto *et al.*, 2006) but not transcript (Song *et al.*, 2008) levels in different studies. However, the small sample numbers and innate variation in p21^CIP1^ levels in these studies does not render such observations inconsistent with ours.

Mimura & Joyce (2006), on the basis of their failure to detect changes in telomere length with HCEC ageing *in vivo*, proposed that entry into senescence in HCEC is telomere independent. We have conducted an interventional test of this hypothesis through ectopic expression of hTERT and have shown that it does not extend lifespan. The Joyce group (Joyce *et al.*, 2011) have also recently shown that the capacity of HCEC to establish primary cultures and proliferate *in vitro* correlates with the amount of oxidative DNA damage received by the cells *in vivo* prior to explants, suggesting that the capacity of HCEC to proliferative is significantly limited by oxidative damage to DNA. We have shown that interventions that manipulate the levels of pro-oxidants (e.g., reduced ppO_2_ and the addition of ascorbic acid 2-phosphate) lengthen replicative lifespan, but that this does not produce immortalization even when telomere erosion is blocked using ectopic hTERT. However, when telomerase expression is combined with either abrogation of p53 or CDK4 over-expression, we find that HCEC reproducibly immortalize. Given the evidence that innate telomere-based protective responses exist that act to reduce oxidative damage to cells (Lee *et al.* 2009), and that the catalytic subunit of telomerase may itself be protective against oxidative damage by a mode of action independent of its effects on the telomere (Ahmed *et al.* 2008), the failure to immortalize with a combination of telomerase and antioxidants appears incompatible with a simple model based on a linear relationship between the accumulation of DNA damage and the onset of replicative senescence in HCEC. The exact nature of the second parallel signal(s) that act to limit HCEC proliferation in concert with telomere erosion is unclear, but could be dissected through additional studies using HCEC cultured under different conditions, and with different proliferative lifespan barriers ablated.

However, from a practical perspective, our key finding is the identification, through interventional approaches, of the role of the p53 pathway, the telomere pathway, and the CDK4/pRb axis in the control of senescence in HCEC. Corneal transplants have a high success rate, but the short supply of donor corneas with competent endothelium (Engelmann *et al.*, 2004) and the continued risk of graft failure because of immunological rejection (Chong & Dana, 2008) remain challenges that may be addressed by the development of bioengineered corneal grafts. In addition, standardized *in vitro* systems containing cultured corneal cells could provide appropriate replacements for routine tests in which animal corneas are commonly used, such as drug permeation studies (Reichl *et al.*, 2004). Hence, there would be great potential utility in the development of human corneal cell lines that are sufficiently differentiated to meet these needs.

Towards this aim, our study demonstrates that it is possible to produce immortalized HCEC that retain differentiated function. Our data show that in addition to retaining the ability to exit the cell cycle in response to contact inhibition, immortalized HCEC-CDK4-hTERT cells retain a HCEC transcriptional profile (Fig. S6), as well as the temperature-sensitive potassium channel that is a key marker for HCEC (Rae & Watsky, 1996), considerably longer than would be required to generate sufficient numbers of cells for most applications. Although the specific cell lines that we have generated would not be good candidates for immediate human *in vivo* studies, they may have significant utility for research on the biology of this cell type, or use in genotoxicology and drug delivery studies.

## Experimental methods

### Cells and cell culture

Primary HCEC, strain 288/97, were cultured in medium F99 comprising a 1:1 mixture of Ham’s F12 (Invitrogen, Paisley, UK) and M199 (Invitrogen) supplemented with 5% foetal calf serum (FCS), 100 μm sodium l-ascorbate (Sigma-Aldrich Company Ltd., Poole, UK), 20 μg mL^−1^ bovine insulin (Sigma-Aldrich Company Ltd.), 2.5 μg mL^−1^ transferrin (Sigma-Aldrich Company Ltd.), 40 pm sodium selenite (Sigma-Aldrich Company Ltd.) and 10 ng mL^−1^ basic fibroblast growth factor (Sigma-Aldrich Company Ltd.). Cells were cultured on plastic, coated using a solution of 5 mg mL^−1^ chondroitin sulphate (Sigma-Aldrich Company Ltd.) and 5 μg mL^−1^ mouse laminin (Invitrogen). They were maintained at 37 °C in a humidified atmosphere containing 5% CO_2_, 95% air unless otherwise specified and re-fed three times a week. Cellular lifespan was expressed in PD, calculated using the standard formula. To study the effects of reducing oxidative stress, HCEC were cultured as above but in 3% O_2_ and the sodium ascorbate in the medium was replaced with 400 μg mL^−1^l-ascorbic acid 2-phosphate magnesium salt (WAKO Biochemicals, Osaka, Japan). For the p38 MAPK inhibition experiment, experimental and control cultures were re-fed daily with medium F99 containing either 10 μm SB203580 (Tocris Bioscience, Bristol, UK) in DMSO or DMSO only, respectively. Quiescent cultures were obtained for the transcriptional profiling experiment by seeding the cells at high density and allowing them to grow to confluency, followed by replacing the medium with F99 containing 0.5% FCS for 3 days before harvesting. Exit from the cell cycle was determined by loss of Ki67 staining.

### Retroviral gene transfer

Amphotropic retrovirus vectors expressing HPV16 E6 genes from a pLXSN construct, packaged in PA317 cells, were kindly provided by Denise Galloway, Seattle, Washington, USA. Human wild-type CDK4 cDNA in pBluescript, kindly given by Gordon Peters, CRUK LRI, London, was recloned into pLXSNneo. hTERT cDNA in pGRN121, a gift from Geron Corp, Menlo Park, CA, USA, was inserted in pBABE retroviral vectors with puro or neo selection. Empty pBABEneo or pBABEpuro vectors packaged in ψCrip cells were used as controls. shRNA targeted to p53 was expressed from pMKO.1PS, kindly donated by William Hahn, Harvard Medical School, packaged in ψCrip. Empty pMLKO.1PS was used as the puromycin control. HCEC for retroviral infection were plated in 6-cm Petri dishes 24 h prior to infection at a seeding density of 6000 cells cm^−2^. Prior to infection, cells were treated with polybrene at 8 μg mL^−1^ for 1 h. Cells were infected by treating with 2 mL of filtered retroviral supernatants supplemented with 8 μg mL^−1^ polybrene for 2 h, followed by the addition of 2 mL F99 and incubation for a further 24 h before replacing the supernatant with 5 mL of fresh F99 medium without selection. Forty-eight hours after the start of infection, HCEC were passaged into fresh dishes with selective medium and cultured as described above to produce bulk cultures of mixed clones.

### RNA isolation and microarray processing

Cells were rinsed briefly with phosphate-buffered saline and then lysed *in situ* using TRIzol (Invitrogen) as per the manufacturer’s instructions. Integrity of the total RNA was confirmed by spectrophotometry and using an Agilent 2100 Bioanalyzer (Agilent Technologies UK Ltd, Edinburgh, UK). Fifteen microgram of labelled cRNA was prepared, essentially as per the standard Affymetrix protocol, from 10 μg of total RNA, using the Superscript II system (Invitrogen) and BioArray High Yield Kit (Enzo Life Sciences Ltd., Exeter, UK), and then hybridized to U133A GeneChips. Expression values and absent/present calls were calculated using mas 5.0 (Affymetrix), using default parameters and a TGT of 100.

### Ki67 immunodetection

Cells on coverslips were fixed with methanol/acetone, 1:1, for 5 min then incubated with a one in 20 dilution of mouse anti-human Ki67 antibody (DAKO UK Ltd., Ely, UK) in 0.1% FCS/PBS at 4 °C overnight. Coverslips were washed and incubated with a one in 30 dilution of rabbit anti-mouse IgG FITC-conjugated antibody (DAKO UK Ltd.) in 0.1% FCS/PBS in the dark for 60 min. Nuclei were counterstained 4′6-diamidino-2-phenylindole (DAPI; Vector Laboratories Ltd., Peterborough, UK). One thousand DAPI-stained cells were counted and the labelling index estimated as the proportion of those cells that stained positively for Ki67.

### Immunoblotting

Cells were lysed for 5 min at 4 °C by 1% NP-40 in 150 mm NaCl–50 mm Tris (pH 8.0)–5 mm EDTA buffer, containing protease inhibitor cocktail III and phosphatase inhibitor cocktail II (Calbiochem, Merck Chemicals Ltd., Nottingham, UK). Protein concentration was estimated using the Coomassie Plus Protein Assay Reagent (Thermo Fisher Scientific, Cramlington, UK). Protein samples were separated by SDS-PAGE and transferred to Immobilon-P polyvinylidene difluoride membrane (Millipore UK Ltd., Watford, UK). Primary antibodies used were anti-p53 mouse monoclonal (DO-1; Calbiochem) diluted 1:1000; anti-CDK4 rabbit polyclonal (C-22; Santa Cruz Biotechnology, Inc., Santa Cruz, CA, USA) diluted 1:800; anti-p16 mouse monoclonal (G175-1239; BD Pharmingen, Oxford, UK) diluted 1/1000. Equal loading was confirmed by detection of α-actin with anti-actin rabbit polyclonal (Sigma-Aldrich Company Ltd.) at a 1:1000 dilution. Bands were visualized by the ECL Plus chemiluminescent detection kit (GE Healthcare, Little Chalfont, UK) using the secondary antibodies provided.

### Electrophysiological measurements

Cells were bathed in HEPES Ringer solution (136 mm NaCl, 2.6 mm CaCl_2_, 2.4 mm KCl, 1.2 mm MgCl_2_, 15 mm HEPES, 10 mm glucose, pH 7.4). The pipette solution was 110 mm KCl, 3.0 mm MgCl_2_, 40 mm HEPES, 3 mm EGTA, pH 7.4. Voltage-gated macroscopic currents were recorded from single cells by a whole-cell patch-clamp technique as described previously (Faragher *et al.*, 1997a). Temperature-sensitive currents were investigated by stepping from a holding potential of −5 mV to between −65 and +75 mV in 10 mV steps for a 500 ms duration at 22 or 34 °C.

## Supporting information

**Fig. S1** Growth curve of a typical primary HCEC culture.

**Fig. S2** Up-regulation of p53 transcriptional targets in senescent HCEC.

**Fig. S3** Lack of effect of p38MAPK inhibitor on growth of primary HCEC.

**Fig. S4** Knockdown of p53 in HCEC by shRNA.

**Fig. S5** Expression of E2F target genes is maintained and elevated in HCEC-CDK4 and HCEC-CDK4-TERT cells.

**Fig. S6** (a) Immortalised HCEC-CDK4-TERT (Zante) cells preserve a similar transcriptional profile to parent primary HCEC cultures. (b) Expression of HCEC-specific transcripts is largely retained in the immortalised HCEC-CDK4-TERT (Zante) cell line.

**Table S1** Over-represented transcription factors by enrichment of their target genes in a cluster of genes up-regulated at senescence in HCEC.

**Table S2** HCEC-specific transcripts.Thirty-three genes identified by microarray analysis (Kipling *etal.*, 2009) of which transcripts were differentiallyup-regulated in proliferating primary HCEC compared with culturesof human diploid fibroblasts (WI38 and HCA2) and primary humanocular keratocytes.

As a service to our authors and readers, this journal provides supporting information supplied by the authors. Such materials are peer-reviewed and may be re-organized for online delivery, but are not copy-edited or typeset. Technical support issues arising from supporting information (other than missing files) should be addressed to the authors.
