# Supplementary material for: Characterization of cellular senescence mechanisms in human corneal endothelial cells
Source: Aging Cell. 2012 Apr;11(2):234–40. doi: 10.1111/j.1474-9726.2011.00776.x (PMC3440103; doi:10.1111/j.1474-9726.2011.00776.x)
Supplement: Supplementary file 5 [file acel0011-0234-SD5.pdf]

a

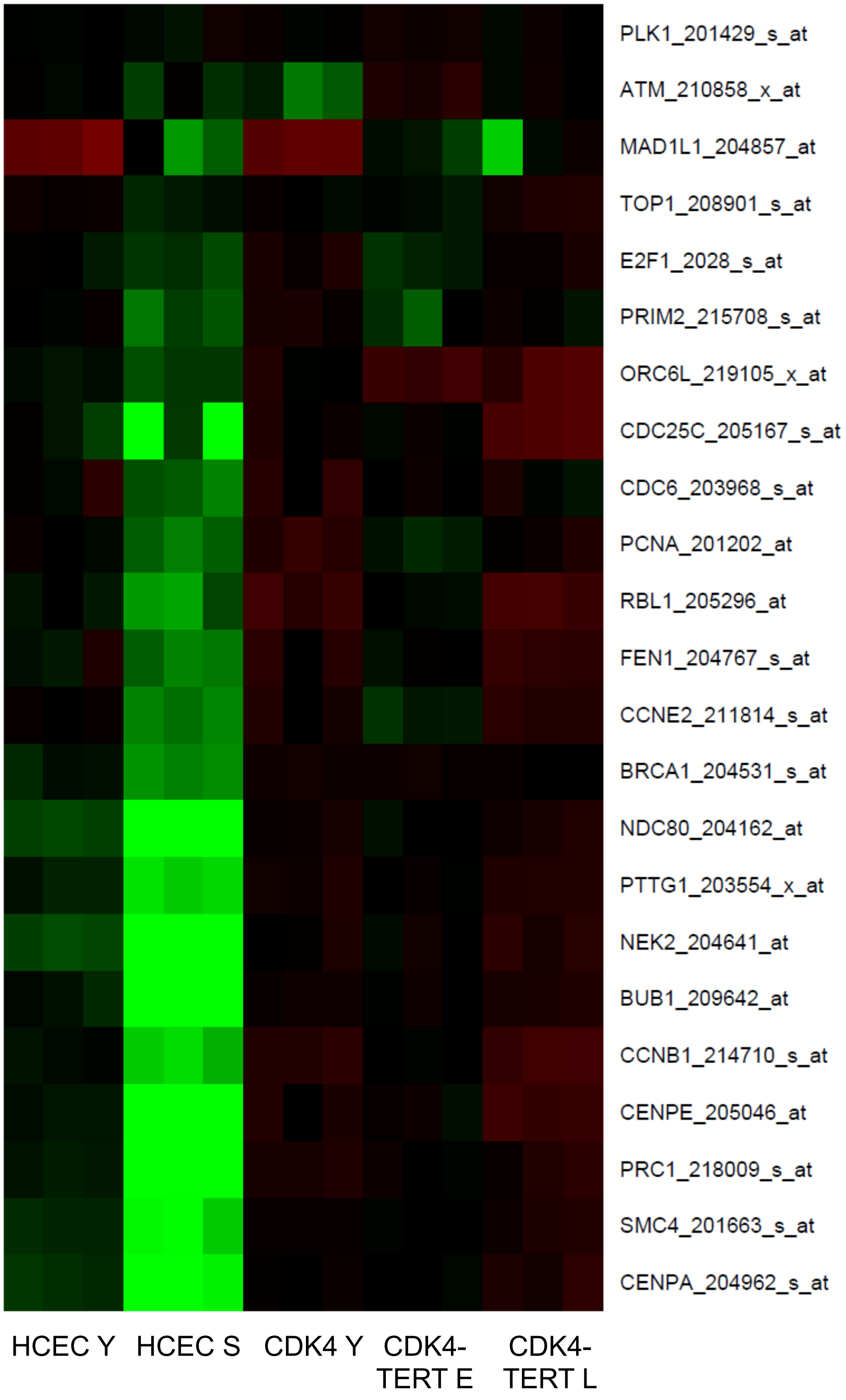

PLK1_201429_s_at
ATM_210858_x_at
MAD1L1_204857_at
TOP1_208901_s_at
E2F1_2028_s_at
PRIM2_215708_s_at
ORC6L_219105_x_at
CDC25C_205167_s_at
CDC6_203968_s_at
PCNA_201202_at
RBL1_205296_at
FEN1_204767_s_at
CCNE2_211814_s_at
BRCA1_204531_s_at
NDC80_204162_at
PTTG1_203554_x_at
NEK2_204641_at
BUB1_209642_at
CCNB1_214710_s_at
CENPE_205046_at
PRC1_218009_s_at
SMC4_201663_s_at
CENPA_204962_s_at
HCEC Y
HCEC S
CDK4 Y
CDK4-TERT E
CDK4-TERT L

b

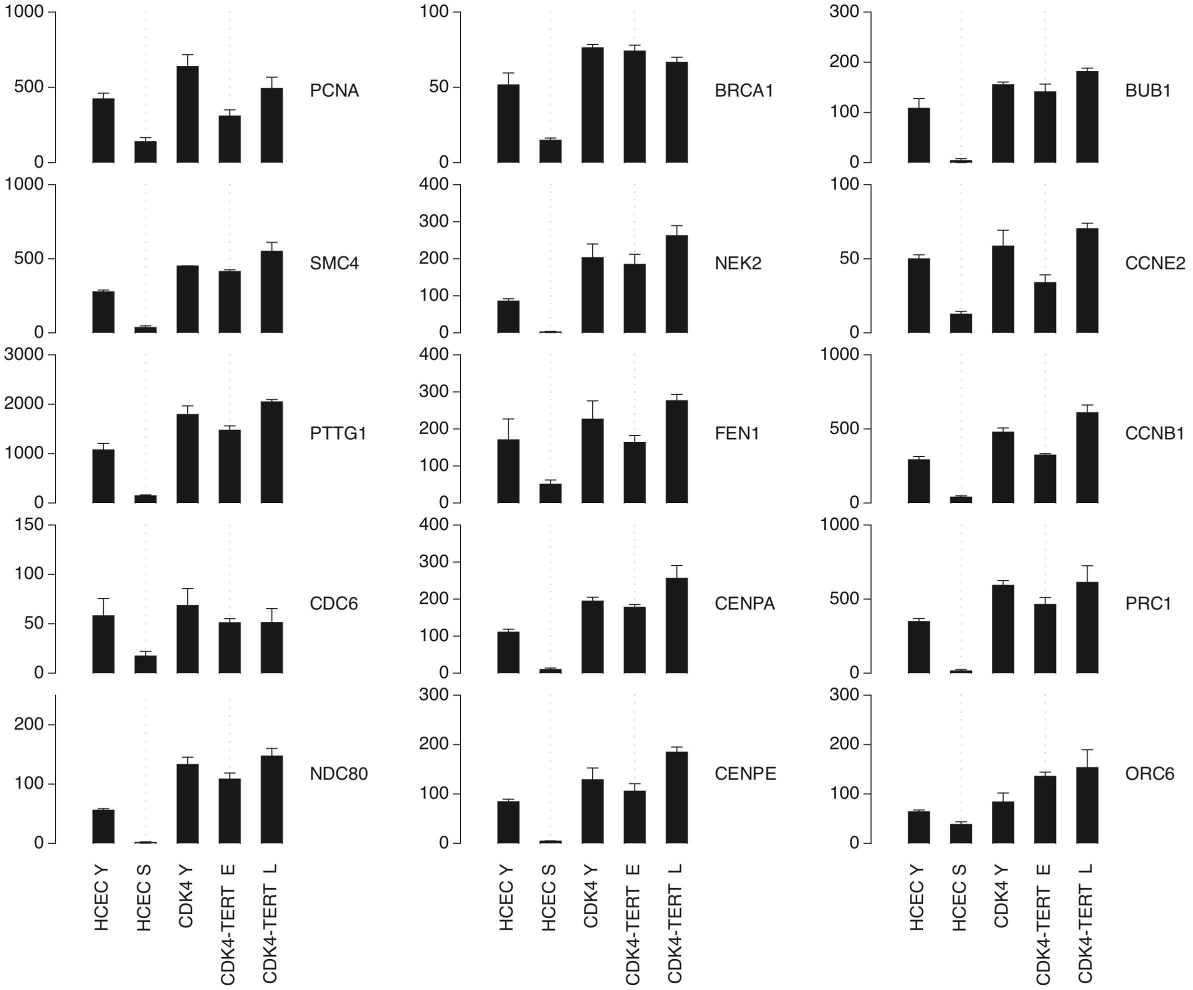
PCNA
BRCA1
BUB1
SMC4
NEK2
CCNE2
PTTG1
FEN1
CCNB1
CDC6
CENPA
PRC1
NDC80
CENPE
ORC6
HCEC Y
HCEC S
CDK4 Y
CDK4-TERT E
CDK4-TERT L

c

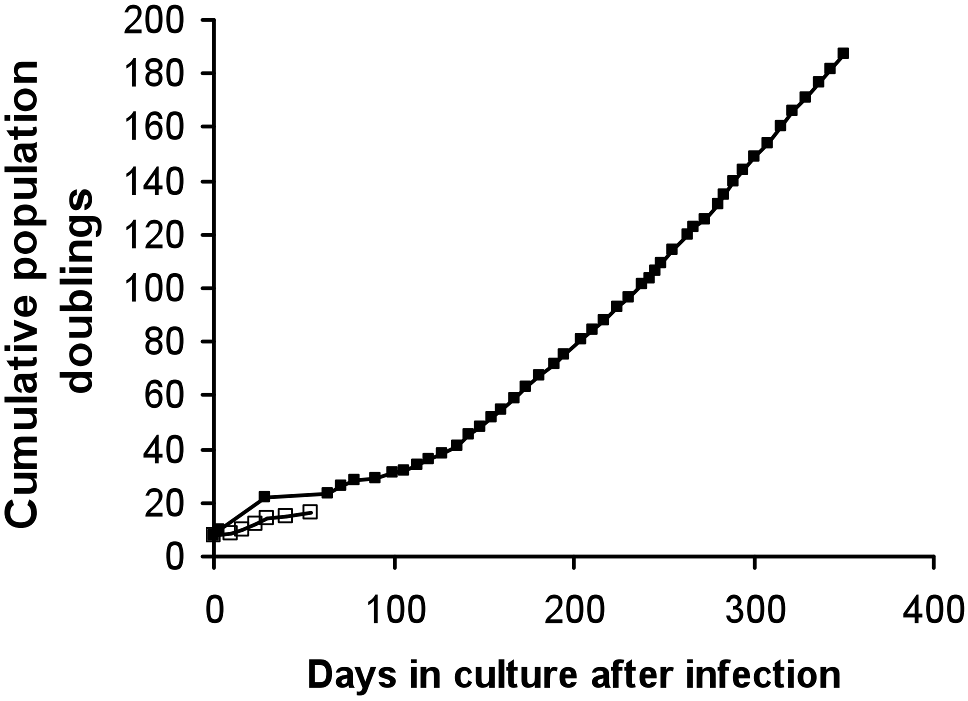
Cumulative population doublings
200
180
160
140
120
100
80
60
40
20
0
0
100
200
300
400
Days in culture after infection

Figure S5 Legend

Expression of E2F target genes is maintained and elevated in HCEC-CDK4 and HCEC-CDK4-TERT cells

Microarray data (see Experimental Methods) was used to examine the expression of a series of previously-defined E2F transcriptional targets (Blais & Dynlacht 2004) in young primary HCEC (HCEC Y; PD10), senescent primary HCEC (HCEC S: PD29), HCEC-CDK4 (CDK4 Y; PD38), and immortalised HCEC-CDK4-TERT cells at PD53 (CDK4-TERT E) and PD193 (CDK4-TERT L). The colour scheme in **a)** indicates expression in log2-space for each probeset relative to the experiment-wide (i.e. row) median such that red indicates upregulation and green indicates lower expression relative to the median. In **b)** the error bars show the standard deviation of *n*=3 samples.

Forced expression of exogenous CDK4 and hTERT by simultaneous infection resulted in an immortal HCEC line

HCEC strain 288/97 was infected at a replicative age of 8 population doublings with retroviral vectors containing human CDK4 and TERT genes respectively and cultured as described in Experimental Methods. In **c)** the chart shows the growth curve of the bulk culture. ■ HCEC-CDK4-hTERT; □ empty vector control.
