# Supplementary material for: Characterization of cellular senescence mechanisms in human corneal endothelial cells
Source: Aging Cell. 2012 Apr;11(2):234–40. doi: 10.1111/j.1474-9726.2011.00776.x (PMC3440103; doi:10.1111/j.1474-9726.2011.00776.x)
Supplement: Supplementary file 6 [file acel0011-0234-SD6.pdf]

a

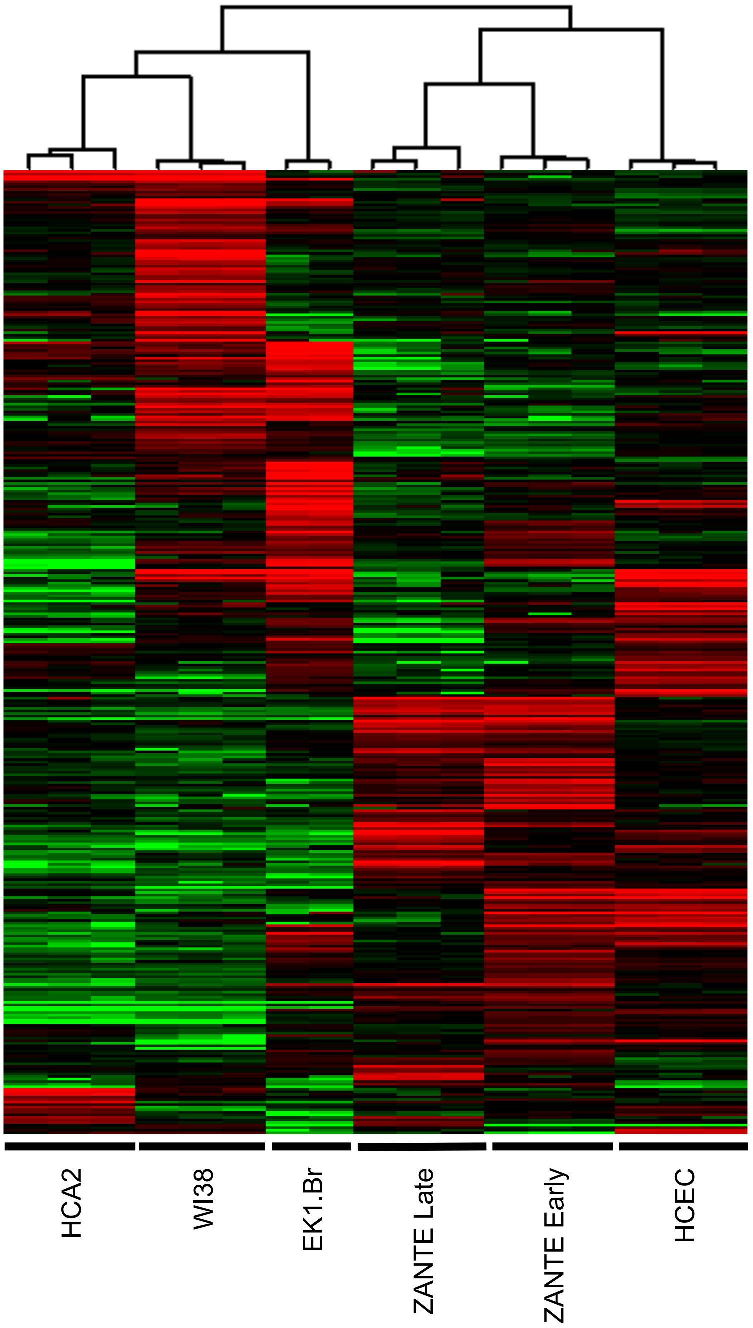

b

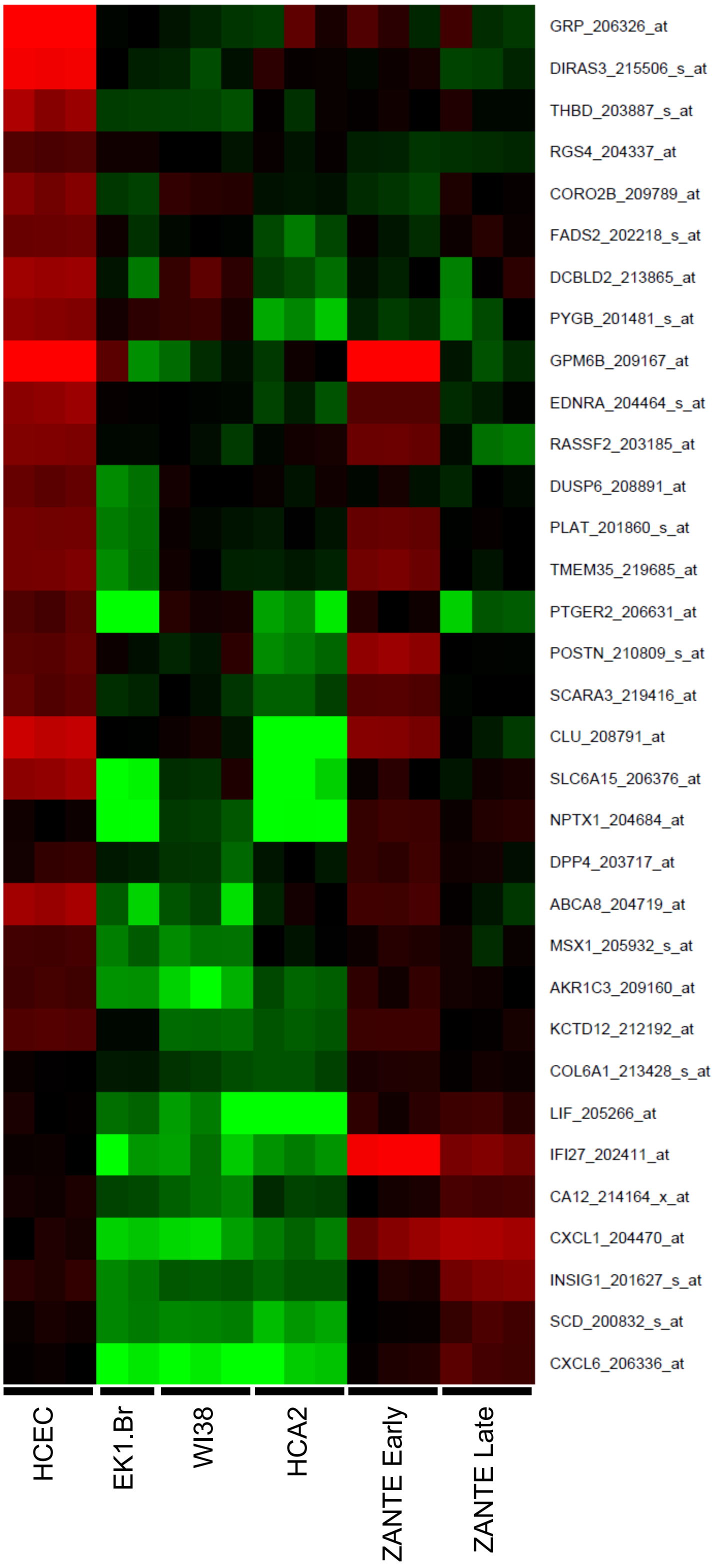
GRP_206326_at
DIRAS3_215506_s_at
THBD_203887_s_at
RGS4_204337_at
CORO2B_209789_at
FADS2_202218_s_at
DCBLD2_213865_at
PYGB_201481_s_at
GPM6B_209167_at
EDNRA_204464_s_at
RASSF2_203185_at
DUSP6_208891_at
PLAT_201860_s_at
TMEM35_219685_at
PTGER2_206631_at
POSTN_210809_s_at
SCARA3_219416_at
CLU_208791_at
SLC6A15_206376_at
NPTX1_204684_at
DPP4_203717_at
ABCA8_204719_at
MSX1_205932_s_at
AKR1C3_209160_at
KCTD12_212192_at
COL6A1_213428_s_at
LIF_205266_at
IFI27_202411_at
CA12_214164_x_at
CXCL1_204470_at
INSIG1_201627_s_at
SCD_200832_s_at
CXCL6_206336_at
HCEC
EK1.Br
WI38
HCA2
ZANTE Early
ZANTE Late

Figure S6 Legend

a) Immortalised HCEC-CDK4-TERT (Zante) cells preserve a similar transcriptional profile to parent primary HCEC cultures

Gene expression profiles were compared from early passage proliferating cultures of primary HCEC, corneal keratocytes (EK1.Br), two strains of human diploid fibroblasts (HCA2 and WI38), and HCEC-CDK4-TERT at PD52 (Zante Early) and PD193 (Zante Late). Differentially expressed genes were discovered by ANOVA. The heatmap shows hierarchical clustering of the top 377 significantly differentially expressed probesets (FDR < 1 x 10^{-6}). The colour scheme indicates expression in $\log_2$-space for each probeset relative to the experiment-wide (i.e. row) median. Red indicates upregulation relative to the row median and green indicates lower expression relative to the median.

b) Expression of HCEC-specific transcripts is largely retained in the immortalised HCEC-CDK4-TERT (Zante) cell line

Thirty-three genes had previously been identified (Kipling *et al*, 2009, Exp Eye Res *88* 277-285) that exhibited high expression levels in primary HCEC cultures compared with corneal keratocytes (strain EK1.Br) and two human diploid fibroblast strains (WI38 and HCA2), and are listed in Table S2. This 33-gene HCEC signature was used to heatmap visual data from HCEC, fibroblasts, keratocytes, and Zante at early and late stages. The colour scheme indicates expression in $\log_2$-space for each probeset relative to the experiment-wide (i.e. row) median. Red indicates upregulation relative to the row median and green indicates lower expression relative to the median.
