# Supplementary material for: Characterization of cellular senescence mechanisms in human corneal endothelial cells
Source: Aging Cell. 2012 Apr;11(2):234–40. doi: 10.1111/j.1474-9726.2011.00776.x (PMC3440103; doi:10.1111/j.1474-9726.2011.00776.x)
Supplement: Supplementary file 7 [file acel0011-0234-SD7.doc]

### Table S1

| **Transcription factor** | **p-value** | **z-score** |
| --- | --- | --- |
| SP1 | 2.866E-14 | 8.503 |
| RelA (p65 NF-kB subunit) | 2.944E-09 | 7.454 |
| HIF1A | 3.148E-09 | 7.585 |
| CREB1 | 8.059E-08 | 6.138 |
| Androgen receptor | 8.865E-07 | 5.828 |
| Oct-1 | 1.729E-06 | 5.712 |
| **p53** | 1.989E-06 | 5.369 |
| ESR1 (nuclear) | 4.121E-06 | 5.124 |
| NF-kB1 (p50) | 1.167E-05 | 5.628 |
| C/EBPbeta | 2.657E-05 | 4.828 |
| EPAS1 | 3.524E-05 | 5.688 |
| ETS1 | 4.058E-05 | 4.783 |
| TCF7L2 (TCF4) | 7.357E-05 | 5.017 |
| NRF2 | 9.910E-05 | 4.969 |
| ATF-2 | 2.428E-04 | 4.645 |
| GCR-alpha | 2.638E-04 | 4.179 |
| HSF1 | 2.705E-04 | 4.45 |
| SRF | 2.735E-04 | 4.258 |
| GATA-1 | 3.005E-04 | 3.99 |
| HNF4-alpha | 3.217E-04 | 3.695 |
| SMAD1 | 3.247E-04 | 5.298 |
| HNF3-beta | 3.249E-04 | 4.315 |
| HSF4 | 4.582E-04 | 5.993 |
| ERG | 7.297E-04 | 4.775 |
| c-Myc | 7.563E-04 | 3.476 |
| EGR1 | 8.999E-04 | 3.627 |
| NF-kB p65/p65 | 9.474E-04 | 5.358 |
| p63 | 1.081E-03 | 3.832 |
| PEA3 | 1.222E-03 | 3.824 |
| TBX2 | 1.255E-03 | 5.921 |
| SOX4 | 1.439E-03 | 4.042 |
| AP-2B | 1.466E-03 | 4.585 |
| LMX1B | 1.609E-03 | 5.65 |
| c-Myb | 1.638E-03 | 3.605 |
| Progesterone receptor | 1.728E-03 | 3.774 |
| VDR | 1.850E-03 | 3.68 |
| SP3 | 1.872E-03 | 3.437 |
| HSF2 | 2.602E-03 | 3.972 |
| c-Rel (NF-kB subunit) | 2.637E-03 | 3.334 |
| STAT5B | 2.785E-03 | 3.93 |
| c-Jun | 2.794E-03 | 3.247 |
| AP-4 | 2.798E-03 | 3.395 |
| NRSF | 3.037E-03 | 3.324 |
| PPAR-gamma | 3.575E-03 | 3.641 |
| ZNF148 | 3.586E-03 | 3.962 |
| AP-2A | 4.505E-03 | 3.093 |
| ATF-4 | 4.513E-03 | 3.804 |
| ARNT | 4.610E-03 | 3.615 |
| CNBP | 4.966E-03 | 5.694 |
| SMAD5 | 5.017E-03 | 4.485 |
| DEC1 (Stra13) | 6.859E-03 | 3.518 |
| SIM2 | 6.866E-03 | 5.22 |

### Table S1 Legend

### Over-represented transcription factors by enrichment of their target genes in a cluster of genes up-regulated at senescence in HCEC.

RNA samples from triplicate cultures of young proliferating (Y; at 10PD) serum-depleted, contact-inhibited quiescent (Q; at 14PD), and serum-starved senescent (S; 29 PD) HCEC were probed with Affymetrix HG-U133A microarrays as described in Experimental Methods, and expression summaries were obtained using the MAS5.0 algorithm.

Differentially expressed transcripts were detected by ANOVA with an FDR of 1% followed by *k*-means clustering and visualisation by heatmapping (see also Supp Fig 3a). Cluster 6 (Fig. S2a) contains 494 transcripts that are differentially upregulated in S HCEC relative to Y & Q HCEC. This cluster was the subject of further analysis and the remaining clusters were not analysed further in this study.

Over-represented transcription factors (TF) in Cluster 6 were identified by enrichment of their respective transcriptional targets in comparison with the MetaCore database (version 6.7; GeneGo, Inc., St. Joseph, MI, USA) using the hypergeometric distribution. The targets of several TF, including p53 (p = 1.989 x 10^-6^), were found to be over-represented by this method.
