# Supplementary material for: Characterization of cellular senescence mechanisms in human corneal endothelial cells
Source: Aging Cell. 2012 Apr;11(2):234–40. doi: 10.1111/j.1474-9726.2011.00776.x (PMC3440103; doi:10.1111/j.1474-9726.2011.00776.x)
Supplement: Supplementary file 8 [file acel0011-0234-SD8.pdf]

Elevated expression retained in immortalised HCEC-CDK4-TERT?	Probe Set ID	Gene Symbol	Gene Title	GO Biological Process	GO Molecular Function	GO Cellular Component
Y	204719_at	ABCA8	ATP-binding cassette, sub-family A (ABC1), member 8	transport /// transport	nucleotide binding /// ATP binding /// ATPase activity /// nucleoside-triphosphatase activity /// ATPase activity, coupled to transmembrane movement of substances	plasma membrane /// membrane /// integral to membrane
Y	209160_at	AKR1C3	aldo-keto reductase family 1, member C3 (3-alpha hydroxysteroid dehydrogenase, type II)	prostaglandin metabolic process /// oxidation reduction /// oxidation reduction	aldo-keto reductase activity /// oxidoreductase activity /// prostaglandin-F synthase activity /// 3-alpha-hydroxysteroid dehydrogenase (A-specific) activity /// testosterone 17-beta-dehydrogenase (NADP+) activity /// trans-1,2-dihydrobenzene-1,2-diol dehydrogenase activity /// indanol dehydrogenase activity /// testosterone 17-beta-dehydrogenase activity	intracellular /// cytoplasm
Y	214164_x_at	CA12	carbonic anhydrase XII	one-carbon metabolic process	carbonate dehydratase activity /// carbonate dehydratase activity /// zinc ion binding /// zinc ion binding /// lyase activity /// metal ion binding	membrane /// integral to membrane /// integral to membrane
Y	208791_at	CLU	clusterin	release of cytochrome c from mitochondria /// lipid metabolic process /// apoptosis /// anti-apoptosis /// response to stress /// complement activation /// complement activation, classical pathway /// response to oxidative stress /// cell death /// positive regulation of cell proliferation /// response to wounding /// response to virus /// endocrine pancreas development /// negative regulation of protein homooligomerization /// negative regulation of apoptosis /// reverse cholesterol transport /// innate immune response /// positive regulation of cell differentiation /// neuron projection morphogenesis /// response to misfolded protein	protein binding /// protein binding /// misfolded protein binding	extracellular region /// extracellular space /// mitochondrion /// aggresome /// extracellular matrix /// spherical high-density lipoprotein particle /// perinuclear region of cytoplasm
Y	213428_s_at	COL6A1	collagen, type VI, alpha 1	cell adhesion /// cell adhesion	platelet-derived growth factor binding	extracellular region /// proteinaceous extracellular matrix /// collagen type VI /// extracellular matrix /// extracellular matrix /// sarcolemma
Y	204470_at	CXCL1	chemokine (C-X-C motif) ligand 1 (melanoma growth stimulating activity, alpha)	chemotaxis /// inflammatory response /// inflammatory response /// immune response /// signal transduction /// G-protein coupled receptor protein signaling pathway /// nervous system development /// cell proliferation /// negative regulation of cell proliferation /// intracellular signaling pathway /// actin cytoskeleton organization	receptor binding /// cytokine activity /// chemokine activity /// chemokine activity /// enzyme activator activity /// growth factor activity	extracellular region /// extracellular region /// extracellular space /// extracellular space
Y	206336_at	CXCL6	chemokine (C-X-C motif) ligand 6 (granulocyte chemotactic protein 2)	chemotaxis /// chemotaxis /// inflammatory response /// immune response /// signal transduction /// cell-cell signaling	cytokine activity /// chemokine activity /// chemokine activity /// heparin binding	extracellular region /// extracellular region /// extracellular space
Y	213865_at	DCBLD2	discoidin, CUB and LCCL domain containing 2	cell adhesion /// negative regulation of cell growth /// intracellular receptor mediated signaling pathway /// wound healing	---	integral to plasma membrane /// cell surface /// membrane /// integral to membrane
Y	203717_at	DPP4	dipeptidyl-peptidase 4	response to hypoxia /// regulation of T cell mediated immunity /// proteolysis /// cell adhesion /// positive regulation of cell proliferation /// negative regulation of extracellular matrix disassembly /// T cell costimulation /// regulation of cell-cell adhesion mediated by integrin /// T cell activation /// endothelial cell migration /// establishment of localization	protease binding /// aminopeptidase activity /// serine-type endopeptidase activity /// receptor activity /// receptor binding /// protein binding /// collagen binding /// peptidase activity /// serine-type peptidase activity /// serine-type peptidase activity /// dipeptidyl-peptidase activity /// dipeptidyl-peptidase activity /// hydrolase activity /// peptide binding /// protein homodimerization activity /// protein homodimerization activity	extracellular region /// membrane fraction /// soluble fraction /// endoplasmic reticulum /// Golgi apparatus /// plasma membrane /// plasma membrane /// cell surface /// cell surface /// membrane /// integral to membrane /// apical plasma membrane /// apical plasma membrane /// lamellipodium /// cell junction /// endocytic vesicle /// lamellipodium membrane /// cell projection /// membrane raft /// intercellular canaliculus /// invadopodium membrane

Y	204464_s_at	EDNRA	endothelin receptor type A	patterning of blood vessels /// response to hypoxia /// in utero embryonic development /// positive regulation of protein phosphorylation /// smooth muscle contraction /// smooth muscle contraction /// signal transduction /// signal transduction /// G-protein coupled receptor protein signaling pathway /// G-protein coupled receptor protein signaling pathway /// activation of adenylate cyclase activity /// activation of phospholipase C activity /// elevation of cytosolic calcium ion concentration /// elevation of cytosolic calcium ion concentration /// activation of protein kinase C activity by G-protein coupled receptor protein signaling pathway /// heart development /// respiratory gaseous exchange /// respiratory gaseous exchange /// cell proliferation /// cell proliferation /// neural crest cell development /// artery smooth muscle contraction /// glucose transport /// glucose transport /// negative regulation of cAMP biosynthetic process /// vasoconstriction /// vasoconstriction /// response to morphine /// regulation of epithelial cell proliferation /// positive regulation of inflammatory response /// positive regulation of release of sequestered calcium ion into cytosol /// elevation of cytosolic calcium ion concentration involved in G-protein signaling coupled to IP3 second messenger /// positive regulation of calcium ion transport	endothelin-A receptor activity /// endothelin-A receptor activity /// phosphoinositide phospholipase C activity /// signal transducer activity /// receptor activity /// G-protein coupled receptor activity /// endothelin receptor activity /// endothelin receptor activity /// protein binding	plasma membrane /// plasma membrane /// integral to plasma membrane /// membrane /// membrane /// integral to membrane /// T-tubule /// nuclear membrane
Y	202218_s_at	FADS2	fatty acid desaturase 2	lipid metabolic process /// fatty acid biosynthetic process /// unsaturated fatty acid biosynthetic process /// transport /// lipid biosynthetic process /// electron transport chain /// oxidation reduction	stearoyl-CoA 9-desaturase activity /// iron ion binding /// oxidoreductase activity /// oxidoreductase activity, acting on paired donors, with oxidation of a pair of donors resulting in the reduction of molecular oxygen to two molecules of water /// heme binding	membrane fraction /// endoplasmic reticulum /// endoplasmic reticulum membrane /// integral to plasma membrane /// membrane /// integral to membrane
Y	209167_at	GPM6B	glycoprotein M6B	multicellular organismal development /// nervous system development /// nervous system development /// cell differentiation	---	membrane /// integral to membrane /// integral to membrane
Y	202411_at	IFI27	interferon, alpha-inducible protein 27	apoptosis /// activation of caspase activity /// induction of apoptosis by extracellular signals /// activation of pro-apoptotic gene products	---	mitochondrion /// mitochondrion /// membrane /// integral to membrane /// integral to membrane
Y	201627_s_at	INSIG1	insulin induced gene 1	lipid metabolic process /// triglyceride metabolic process /// cholesterol biosynthetic process /// response to sterol depletion /// metabolic process /// steroid metabolic process /// cholesterol metabolic process /// cell proliferation /// negative regulation of steroid biosynthetic process /// sterol biosynthetic process /// ER-nuclear sterol response pathway /// inner ear morphogenesis /// middle ear morphogenesis /// negative regulation of fat cell differentiation /// negative regulation of fatty acid biosynthetic process /// palate development /// cranial suture morphogenesis	protein binding	endoplasmic reticulum /// endoplasmic reticulum /// endoplasmic reticulum membrane /// membrane /// integral to membrane
Y	212192_at	KCTD12	potassium channel tetramerisation domain containing 12	potassium ion transport	voltage-gated potassium channel activity /// protein binding /// protein binding	plasma membrane /// voltage-gated potassium channel complex /// membrane /// cell junction /// presynaptic membrane /// synapse /// postsynaptic membrane

Y	205266_at	LIF	leukemia inhibitory factor (cholinergic differentiation factor)	blood vessel remodeling /// immune response /// multicellular organismal development /// embryo implantation /// positive regulation of cell proliferation /// positive regulation of cell proliferation /// positive regulation of cell proliferation /// negative regulation of cell proliferation /// positive regulation of gene-specific transcription from RNA polymerase II promoter /// stem cell maintenance /// lung development /// organ regeneration /// positive regulation of peptidyl-serine phosphorylation /// tyrosine phosphorylation of Stat3 protein /// positive regulation of tyrosine phosphorylation of Stat3 protein /// positive regulation of MAPKKK cascade /// regulation of cell differentiation /// positive regulation of macrophage differentiation /// negative regulation of meiosis /// positive regulation of transcription from RNA polymerase II promoter /// decidualization /// negative regulation of hormone secretion /// lung alveolus development /// muscle organ morphogenesis /// neuron development /// positive regulation of astrocyte differentiation /// leukemia inhibitory factor signaling pathway /// positive regulation of peptidyl-tyrosine phosphorylation /// maternal process involved in female pregnancy /// lung vasculature development /// lung lobe morphogenesis /// trophoblast giant cell differentiation /// spongiotrophoblast differentiation /// negative regulation of ERK1 and ERK2 cascade	receptor binding /// cytokine activity /// cytokine activity /// leukemia inhibitory factor receptor binding /// leukemia inhibitory factor receptor binding /// leukemia inhibitory factor receptor binding /// growth factor activity /// growth factor activity	extracellular region /// extracellular space /// extracellular space
Y	205932_s_at	MSX1	msh homeobox 1	negative regulation of transcription from RNA polymerase II promoter /// skeletal system development /// in utero embryonic development /// epithelial to mesenchymal transition /// heart morphogenesis /// transcription /// regulation of transcription, DNA-dependent /// multicellular organismal development /// heart development /// muscle organ development /// negative regulation of cell proliferation /// organ morphogenesis /// mesenchymal cell proliferation /// positive regulation of gene-specific transcription from RNA polymerase II promoter /// embryonic limb morphogenesis /// BMP signaling pathway /// forebrain development /// midbrain development /// odontogenesis /// negative regulation of apoptosis /// regulation of transcription /// negative regulation of transcription, DNA-dependent /// positive regulation of transcription from RNA polymerase II promoter /// cartilage development /// response to protein stimulus /// cellular response to protein stimulus	DNA binding /// sequence-specific DNA binding transcription factor activity /// protein binding /// transcription repressor activity /// transcription regulator activity /// sequence-specific DNA binding	nucleus
Y	204684_at	NPTX1	neuronal pentraxin I	transport /// synaptic transmission /// central nervous system development	metal ion binding	transport vesicle /// cytoplasmic vesicle
Y	201860_s_at	PLAT	plasminogen activator, tissue	response to hypoxia /// protein modification process /// proteolysis /// proteolysis /// blood coagulation /// smooth muscle cell migration /// synaptic transmission, glutamatergic /// response to peptide hormone stimulus /// negative regulation of proteolysis /// platelet-derived growth factor receptor signaling pathway /// regulation of synaptic plasticity /// blood vessel morphogenesis /// response to glucocorticoid stimulus /// response to cAMP	catalytic activity /// serine-type endopeptidase activity /// serine-type endopeptidase activity /// serine-type endopeptidase activity /// protein binding /// peptidase activity /// serine-type peptidase activity /// hydrolase activity	extracellular region /// extracellular region /// extracellular space /// cytoplasm /// cytoplasm /// cell surface /// stored secretory granule /// extracellular matrix /// apical part of cell /// synapse
Y	210809_s_at	POSTN	periostin, osteoblast specific factor	skeletal system development /// cell adhesion /// cell adhesion	heparin binding /// heparin binding	extracellular region /// proteinaceous extracellular matrix /// proteinaceous extracellular matrix /// extracellular matrix

Y	206631_at	PTGER2	prostaglandin E receptor 2 (subtype EP2), 53kDa	signal transduction /// G-protein coupled receptor protein signaling pathway /// G-protein coupled receptor protein signaling pathway /// regulation of cell proliferation	signal transducer activity /// receptor activity /// G-protein coupled receptor activity /// prostaglandin E receptor activity /// prostaglandin E receptor activity	plasma membrane /// plasma membrane /// integral to plasma membrane /// membrane /// integral to membrane
Y	203185_at	RASSF2	Ras association (RalGDS/AF-6) domain family member 2	cell cycle /// signal transduction	protein binding /// protein binding	nucleus
Y	219416_at	SCARA3	scavenger receptor class A, member 3	response to oxidative stress /// UV protection	scavenger receptor activity	Golgi membrane /// endoplasmic reticulum /// endoplasmic reticulum membrane /// Golgi apparatus /// membrane /// integral to membrane
Y	200832_s_at	SCD	stearoyl-CoA desaturase (delta-9-desaturase)	lipid metabolic process /// fatty acid biosynthetic process /// lipid biosynthetic process /// oxidation reduction	stearoyl-CoA 9-desaturase activity /// stearoyl-CoA 9-desaturase activity /// iron ion binding /// oxidoreductase activity /// oxidoreductase activity, acting on paired donors, with oxidation of a pair of donors resulting in the reduction of molecular oxygen to two molecules of water	endoplasmic reticulum /// endoplasmic reticulum /// endoplasmic reticulum membrane /// membrane /// integral to membrane /// nuclear membrane
Y	219685_at	TMEM35	transmembrane protein 35	---	---	membrane /// integral to membrane
N	209789_at	CORO2B	coronin, actin binding protein, 2B	actin cytoskeleton organization	actin binding /// actin binding	cytoplasm /// cytoskeleton /// actin cytoskeleton /// membrane
N	215506_s_at	DIRAS3	DIRAS family, GTP-binding RAS-like 3	regulation of cyclin-dependent protein kinase activity /// regulation of gene expression by genetic imprinting /// signal transduction /// small GTPase mediated signal transduction /// small GTPase mediated signal transduction	nucleotide binding /// GTPase activity /// GTPase activity /// GTP binding	plasma membrane /// membrane
N	208891_at	DUSP6	dual specificity phosphatase 6	inactivation of MAPK activity /// negative regulation of protein phosphorylation /// protein dephosphorylation /// response to organic cyclic substance /// dephosphorylation /// cell differentiation /// peptidyl-tyrosine dephosphorylation /// response to drug /// positive regulation of apoptosis /// response to nitrosative stress /// regulation of heart growth /// negative regulation of ERK1 and ERK2 cascade /// negative regulation of ERK1 and ERK2 cascade /// response to growth factor stimulus	phosphoprotein phosphatase activity /// protein tyrosine phosphatase activity /// protein tyrosine phosphatase activity /// protein binding /// protein tyrosine/serine/threonine phosphatase activity /// hydrolase activity /// phosphatase activity /// MAP kinase tyrosine/serine/threonine phosphatase activity /// MAP kinase tyrosine/serine/threonine phosphatase activity	nucleoplasm /// cytoplasm /// cytoplasm
N	206326_at	GRP	gastrin-releasing peptide	signal transduction /// neuropeptide signaling pathway /// neuropeptide signaling pathway	receptor binding /// neuropeptide hormone activity	extracellular region /// extracellular region /// extracellular space
N	201481_s_at	PYGB	phosphorylase, glycogen; brain	carbohydrate metabolic process /// glycogen metabolic process /// glycogen catabolic process /// glycogen catabolic process	phosphorylase activity /// sugar binding /// drug binding /// glycogen phosphorylase activity /// transferase activity /// transferase activity, transferring glycosyl groups /// pyridoxal phosphate binding /// protein homodimerization activity	soluble fraction /// cytoplasm /// axon
N	204337_at	RGS4	regulator of G-protein signaling 4	inactivation of MAPK activity /// regulation of G-protein coupled receptor protein signaling pathway /// negative regulation of signal transduction	signal transducer activity /// GTPase activator activity /// GTPase activator activity /// calmodulin binding	membrane fraction /// cytoplasm /// cytosol /// plasma membrane
N	206376_at	SLC6A15	solute carrier family 6 (neutral amino acid transporter), member 15	transport /// neurotransmitter transport /// neurotransmitter transport /// amino acid transport	neurotransmitter transporter activity /// neurotransmitter:sodium symporter activity /// amino acid transmembrane transporter activity /// symporter activity	plasma membrane /// integral to plasma membrane /// membrane /// integral to membrane /// integral to membrane
N	203887_s_at	THBD	thrombomodulin	female pregnancy /// blood coagulation /// blood coagulation /// embryo development /// response to X-ray /// negative regulation of platelet activation /// negative regulation of blood coagulation /// response to lipopolysaccharide /// response to cAMP /// negative regulation of fibrinolysis	receptor activity /// receptor activity /// transmembrane receptor activity /// binding /// calcium ion binding /// calcium ion binding /// protein binding	extracellular space /// plasma membrane /// plasma membrane /// integral to plasma membrane /// cell surface /// membrane /// integral to membrane
